# Supplementary material for: Maternal haemodynamics in Hypertensive Disorders of Pregnancy under antihypertensive therapy (HyperDiP): study protocol for a prospective observational case–control study
Source: BMJ Open. 2023 Jun 1;13(6):e065444. doi: 10.1136/bmjopen-2022-065444 (PMC10254794; doi:10.1136/bmjopen-2022-065444)
Supplement: Author's manuscript [file bmjopen-2022-065444.draft_revisions.pdf]

BMJ Open is committed to open peer review. As part of this commitment we make the peer review history of every article we publish publicly available.

When an article is published we post the peer reviewers' comments and the authors' responses online. We also post the versions of the paper that were used during peer review. These are the versions that the peer review comments apply to.

The versions of the paper that follow are the versions that were submitted during the peer review process. They are not the versions of record or the final published versions. They should not be cited or distributed as the published version of this manuscript.

BMJ Open is an open access journal and the full, final, typeset and author-corrected version of record of the manuscript is available on our site with no access controls, subscription charges or pay-per-view fees (http://bmjopen.bmj.com).

If you have any questions on BMJ Open's open peer review process please email info.bmjopen@bmj.com

BMJ Open

Maternal hemodynamics in Hypertensive Disorders of Pregnancy under antihypertensive therapy (HyperDiP): a prospective cohort study

Journal:	*BMJ Open*
Manuscript ID	bmjopen-2022-065444
Article Type:	Protocol
Date Submitted by the Author:	13-Jun-2022
Complete List of Authors:	Palmrich, Pilar; Hospital of the Medical University of Vienna Division of Obstetrics and Feto-Maternal Medicine<br>Haase, Nadine; Experimental and Clinical Research Center, a cooperation between the Max Delbrück Center for Molecular Medicine in the Helmholtz Association and Charité Universitätsmedizin ; Max - Delbrück Center for Molecular Medicine in the Helmholtz Association (MDC<br>Sugulle, Meryam ; Oslo University Hospital, Division of Gynecology and Obstetrics; University of Oslo Faculty of Medicine<br>Kalafat, E. ; Koc University Hospital, Department of Obstetrics and Gynecology,<br>Khalil, Asma; St George's Hospital, Fetal Medicine Unit<br>Binder, Julia; Hospital of the Medical University of Vienna Division of Obstetrics and Feto-Maternal Medicine
Keywords:	Maternal medicine < OBSTETRICS, Ultrasonography, Hypertension < CARDIOLOGY

SCHOLARONE™
Manuscripts

Maternal hemodynamics in Hypertensive Disorders of Pregnancy under antihypertensive therapy (HyperDiP): a prospective cohort study

Pilar Palmrich[1], Nadine Haase[2-5], Meryam Sugulle[6-7], Erkan Kalafat [8], Asma Khalil[9], Julia Binder[1]

[1] Department of Obstetrics and Feto-Maternal Medicine, Medical University of Vienna
[2] Experimental and Clinical Research Center, a cooperation between the Max Delbrück Center for Molecular Medicine in the Helmholtz Association and Charité Universitätsmedizin Berlin, Germany
[3] Max - Delbrück Center for Molecular Medicine in the Helmholtz Association (MDC), Berlin, Germany
[4] Charité – Universitätsmedizin Berlin, corporate member of Freie Universität Berlin, Humboldt-Universität zu Berlin, Experimental and Clinical Research Center, Berlin, Germany
[5] DZHK (German Centre for Cardiovascular Research), partner site Berlin, Germany
[6] Division of Gynecology and Obstetrics, Oslo University Hospital, Ullevål, Oslo, Norway
[7] University of Oslo, Faculty of Medicine, Oslo, Norway
[8] Department of Obstetrics and Gynecology, Koc University Hospital, Istanbul, Turkey
[9] Fetal Medicine Unit, St. George's Hospital St George's University of London, London, United Kingdom

Corresponding author:

Pilar Palmrich, MD

Department of Obstetrics and Feto-Maternal Medicine,

Division of Obstetrics and Gynecology, Medical University of Vienna

Währinger Gürtel 18-20, A-1090 Vienna, Austria

T: +43 1 4040028220, F: +43 1 40400 28620

Email: pilar.palmrich@meduniwien.ac.at

For peer review only

ABSTRACT:

Introduction: Hypertensive disorders of pregnancy (HDP) are associated with a high incidence of maternal and perinatal morbidity and mortality. HDP, in particular preeclampsia, have been determined as risk factors for future cardiovascular disease (CVD). Recently, the common hypothesis of preeclampsia being a placental disorder was challenged as numerous studies show evidence for short- and long-term cardiovascular changes in pregnancies affected by HDP, suggesting a cardiovascular origin of the disease. Despite new insights into the pathophysiology of HDP, concepts of therapy remain unchanged and evidence for improved maternal and neonatal outcome by using antihypertensive agents is lacking.

Methods and Analysis: A prospective observational case control study, including women with HDP and a healthy control group, which will assess maternal hemodynamics using the USCOM©1A Monitor and Arteriograph© along with cardiovascular markers in women with HDP under antihypertensive therapy, including a follow-up at three months and one-year post-partum will be conducted over a 36-month period in Vienna. A prospective, longitudinal study of cardiovascular surrogate markers conducted in Oslo will serve as a comparative cohort for the Vienna cohort of hemodynamic parameters in pregnancy including a longer follow up period of up to 3 years post-partum.

Ethics and Dissemination: Ethical approval has been granted to all participating centers. Results will be published in international peer-reviewed journals and will be presented at national and international conferences.

For peer review only

STRENGTHS AND LIMITATIONS

To the best of our knowledge, this is the first study assessing changes in maternal hemodynamics in combination with cardiovascular markers prior to and after antihypertensive treatment in women with hypertensive disorders of pregnancy compared to a healthy gestational age matched control group. Furthermore, our analysis will include comparison between single and combined oral antihypertensives. We believe that these investigations have the potential to change management strategies in women with antihypertensive disorders of pregnancy and will warrant a tailored treatment approach. A potential limitation of the study worth noting is that the comparison between different groups of antihypertensive agents might pose a challenge as the sample sizes within the respective groups will be small as we assume that a large proportion of patients will receive only one antihypertensive drug.

For peer review only

BACKGROUND

Hypertensive disorders of pregnancy (HDP) are still the leading cause of maternal and perinatal morbidity and mortality worldwide. [1,2] Women undergo marked physiological systemic changes during pregnancy. Regarding the cardiovascular system, pregnancy acts as a cardiac stress model. Despite echocardiographically detectable changes, most pregnant women tolerate the inflicted cardiac stress without development of clinical symptoms. [3] HDP, in particular the pregnancy specific syndrome preeclampsia, have been found to be common risk factors for future cardiovascular disease (CVD), even in previously healthy women without evidence of previous cardiovascular disease. [4,5] The underlying pathophysiology and pathomechanism leading to preeclampsia are not yet fully understood and have been the focus of research in the last decades. Recently, the common hypothesis of preeclampsia being a placental disorder was challenged as a number of studies show evidence for short and long term cardiovascular changes in pregnancies affected by preeclampsia, possibly suggesting a cardiovascular origin of the disease. These cardiovascular changes include impaired cardiac output, an increase in systemic vascular resistance and left ventricular diastolic dysfunction especially in women affected by early onset preeclampsia. [6-10] Studies on maternal hemodynamic changes in women with preeclampsia revealed persistent long-term cardiac alterations of up to two years after preeclampsia and an increased lifetime risk of essential hypertension and cardiovascular disease. [11] Although there are new insights into a potential cardiovascular origin of the disease, the strategies of treating preeclampsia have not changed fundamentally in the last years. Furthermore, there is no international consensus on when and how increased blood pressure in pregnancy should be treated. [12] Studies of maternal hemodynamics in women suffering from HDP on antihypertensive treatment indicate effectiveness of hemodynamically guided antihypertensive therapy. The data to support this, however, are limited. Khalil et al. demonstrated significantly lower arterial stiffness in women treated with alpha-methyldopa, the first line treatment of hypertension in pregnancy [13] in many countries. A study by Stott et al., evaluating antihypertensive therapy with labetalol and nifedipine guided by hemodynamic parameters in pregnant women with HDP, was able to demonstrate a significant decrease in episodes of severe hypertension when treatment was guided by hemodynamic parameters. [14] Studies on non-pregnant women undergoing hemodynamic monitoring while receiving antihypertensive therapy support this approach of a hemodynamically guided therapy when blood pressure is resistant or refractory. [12] Cardiovascular markers such as soluble fms like kinase 1 (sFlt-1) and placental like growth factor (PIGF) have been introduced into clinical practice to help estimating the time to delivery in women with preeclampsia. [15] A study by Sugulle et al. indicated the usefulness of midregional proatrial natriuretic peptide (MR-proANP) as a biomarker in preeclampsia, both in humans and in a rat model, likely to reflect cardiovascular hemodynamic stress in these

For peer review only

women.[16] N-terminal pro-B type natriuretic peptide (NT-pro BNP) is known to be released by cardiomyocytes due to ischemia and increased mechanical strain seen in women with preeclampsia but also in non-pregnant patients with heart failure, showed potential in predicting time to delivery in combination with the sFlt1/PlGF ratio within one week. [17] In a prospective study assessing biophysical and biochemical markers of cardiovascular strain and placental dysfunction in patients with HDP and healthy controls, Verlohren et al. also demonstrated the additive predictive value of total peripheral resistance index (TPRI), with additional moderate contribution of NT-proBNP and cardiac index (CI) in combination with sFlt-1/PlGF ratio for the prediction of HDP. [18]

OBJECTIVES:

This project aims to evaluate cardiovascular parameters in women affected by HDP by assessing maternal hemodynamic function during pregnancy as well as in the postpartum period up until 3 years after delivery. The focus of this study is to evaluate maternal hemodynamics as well as angiogenic and cardiovascular markers (sFlt-1/PlGF, NT-pro BNP) under antihypertensive therapy in women affected by HDP compared to a healthy control group matched for gestational age to assess whether cardiac indices should be used as a guiding tool for antihypertensive therapy in these women. In this study we focus on changes in maternal hemodynamics secondary to antihypertensive treatment, also comparing single and combined oral antihypertensive therapy in women with HDP.

METHODS

Assessment of maternal hemodynamic function in pregnancy and postpartum - Vienna:

This is an observational cohort study over a 36-month period, assessing women with hypertensive disorders of pregnancy (HDP), including both gestational hypertension and preeclampsia, as well as a control group composed of healthy pregnant women matched for gestational age. All women will be recruited at the high-risk maternal outpatient clinic and antenatal ward at the Department of Obstetrics and fetomaternal Medicine at the Medical University of Vienna.

Women fulfilling eligibility criteria (see Box 1 and 2) will undergo non-invasive assessment of maternal hemodynamics including determination of heart rate, cardiac output, cardiac index, stroke volume and systemic vascular resistance using USCOM© 1A Monitor, non-invasive assessment of arterial stiffness using Arteriograph® as well as measurement of sFlt1/PlGF ratio and NTproBNP by taking blood samples at each hemodynamic assessment (figure 1).

For peer review only

Box 1: Inclusion criteria

Pregnant women 20+0 and 42+0 weeks of gestation with a

- singleton pregnancy
- Aged 18 years or older
- Able to give valid informed consent
- Presenting with gestational hypertension or preeclampsia

Box 2: Exclusion criteria

- Major fetal anomaly or aneuploidy/genetic syndrome
- Maternal and fetal cardiac defects
- Systemic cardiovascular diseases (e.g. systemic lupus erythematodes with end organ dysfunction)
- Diabetes mellitus type I and II
- Chronic hypertension
- Chronic kidney disease

Primary and secondary outcome measures are summarized in Box 3 to 5.

Box 3: Primary Outcomes

- **Assessment of maternal hemodynamics in women with hypertensive disorders of pregnancy under antihypertensive therapy:**
  - Improvement of hemodynamic parameters under treatment?
  - Differences in maternal hemodynamic parameters between women treated with different groups of antihypertensive agents?
- **Assessment of angiogenic factors (sFlt-1/PlGF, NTproBNP) in women with HDP under antihypertensive therapy:**
  - Is the improvement of hemodynamic parameters reflected by a decrease in angiogenic factors?

Box 4: Secondary Outcomes: maternal adverse outcome

- Hepatic dysfunction (ALT ≥100IU/L)
- Acute renal insufficiency (defined as creatinine ≥100 micromol/L antenatally, or ≥130 postnatally) or need for dialysis
- Placental abruption (Retroplacental clot or associated with preterm delivery or fetal demise) described clinically
- Admission to the intensive care unit
- Days of hospital admission
- Mode of delivery
- Post-partum hemorrhage (defined as more than >500 ml blood loss after delivery
- Development of chronic hypertension in the follow up period

Box 5: Secondary Outcomes: perinatal/neonatal outcome

- Gestational age at delivery
- Birth weight
- Stillbirth
- Neonatal death
- Neonatal morbidity (admission to neonatal unit for more than 48 hours, respiratory distress syndrome, need for intubation, intraventricular hemorrhage, confirmed infection, necrotizing enterocolitis, seizures, encephalopathy, retinopathy of prematurity)
- Need for admission to Neonatal Intensive Care Unit (NICU)
- Need for admission to Special Care Baby Unit
- Number of bed nights in NICU associated with delivery for PE

USCOM© 1A Monitor is a device using continuous wave Doppler through a non-imaging probe in the suprasternal notch. It assesses velocity time integrals of transaortic blood flow at the left ventricular outflow tract, obtaining a complete hemodynamic profile. The Arteriograph® device is a validated method of non-invasive assessment of arterial stiffness by measuring pulse wave velocity (PWVao), aortic augmentation index (Aix) and central systolic blood pressure (SBPao) using a simple upper arm cuff. Serum sFlt1/PlGF ratio and NT- proBNP will be measured using an automated immune analyzer (Roche Diagnostics, Germany) at each hemodynamic assessment. Initial evaluation, if possible, will take place prior to starting antihypertensive treatment, followed by assessment after 24-72 hours after initiation of antihypertensive therapy. The same measurements will be repeated if therapy is extended by another antihypertensive agent. If no additional antihypertensive therapy is necessary, another

assessment will be performed in the third trimester or prior to delivery if indicated due to critical maternal or neonatal situation. In the postpartum period assessments will be undertaken within one week after delivery, and after a 3 to 6 month and a one year follow up period (see figure 2). Antihypertensive treatment will be initiated as per local protocol, which is based on the updated AWMF (Association of the scientific medical societies in Germany) guideline. [19] The control group will have serial hemodynamic assessment and blood sampling for cardiovascular parameters (as described for the cases group previously) in a four-week interval during pregnancy and within a one-week interval after birth, after 3 to 6 months and one year post-partum.

Sample size

The sample size estimation was done using the G*Power software (version 3.1.9.6, Universität Düsseldorf, Germany). [20] We estimated the sample size assuming a repeated measures ANCOVA model to test the between factor significance with the following parameters; effect size f: 0.15; error probability: 0.05, power: 0.90. We used a small to moderate generic effect size instead of tailoring it to a specific parameter, as a number of cardiovascular parameters will be compared between the groups. We further assumed an average of four measurements per group with a weak correlation between repeated measures (0.2). Under these assumptions, a total sample size of 220 (110 patients in each) was estimated to reach the targeted power level.

Postnatal longitudinal assessment of cardiovascular surrogate markers - Oslo:

Oslo University Hospital, Ullevål will provide a comparative cohort for the Vienna cohort of hemodynamics in pregnancy. The Norwegian "HAPPY" - "Health after pregnancy complications" study, which currently comprises 221 women at 1-year and 108 women at 3 year follow up, among others including women after HDP and control patients, will be able to provide longitudinal data on cardiovascular surrogate markers such as endothelial function, carotid artery wall structure and aortic stiffness. Also, circulating biomarkers for CVD risk, such as systemic inflammatory marker hsCRP, and systemic "metabolic stress" markers leptin, insulin resistance (HoMa score), fasting blood lipids (Triglycerides, LDL-Cholesterol, HDL-C, oxLDL), measured by immunoassays and multiplex assays. [21] Additionally, the Oslo Pregnancy Biobank, contains maternal blood and umbilical blood as well as placental tissue samples for assessment of morphological changes and will add additional comparative data.

For peer review only

Furthermore, the CHASE study "Cardiovascular health after pregnancy complications" will serve as an another postpartum cohort, including blood samples and surrogate markers for endothelial function of 40 mother-child pairs 5 to 8 years postpartum. [22]

Patient and Public Involvement:

Patients or the public will not be involved in the design, conduct, reporting, or dissemination plans of our research.

STATISTICAL ANALYSIS

Each site will provide a unique dataset comprising a patient group, the respective preeclamptic rat model, and a control group. Three sets of analyses are planned for each outcome category (USCOM measurements; Arteriograph measurements; Angiogenic marker measurements).

Baseline differences between hypertensive and normotensive pregnancies

Measurements at the time of enrolment will be used for this analysis. Variables will be described using location and scale parameters (mean, standard deviation, median, interquartile range etc.) appropriate for their distribution characteristics. Groups will be compared after matching for predefined confounders (BMI and gestational age at assessment). Mean differences or odds ratio with 95% confidence intervals will be reported to describe the effect sizes. Effect sizes will be estimated with generalized estimating equations using match identifiers as a cluster.

Estimating the effect of antihypertensive therapy on outcome parameters

Measurements at the time of enrollment and after the initiation of antihypertensive therapy (24 to 72 hours) will be used for this analysis. Paired measurements will be used to estimate the mean differences or odds ratio with 95% confidence intervals. For variables with skewed distributions, median differences and nonparametric bootstrapped confidence intervals will be reported. Also, clinical factors associated with before and after changes will be investigated and will be adjusted if necessary.

Longitudinal change of outcome parameters between hypertensive and normotensive pregnancies

Repeat measurements from normotensive pregnancies and hypertensive pregnancies which were not medicated will be used for this analysis. A two-stage approach will be adopted for analysis. First, generalized linear mixed-effects models (GLMM) with appropriate link function

For peer review only

and random slopes for patient identifiers will be used to model the change of outcome variables with gestational age. Random effects from GLMM models will be extracted and the effect of hypertension status will be tested with linear models. All analyses will be conducted using R for Statistical Computing Software (R foundation, Vienna, Austria) performed by an appointed statistician.

DATA MONITORING

Study participants will be anonymized by assigning a sequential patient number. All data will be entered, processed and stored anonymously in a case report form the online data management program Clincase © by the study investigators.

ETHICS and DISSEMINATION

This study will be conducted in accordance with the principles of Good Clinical Practice (GCP). The local ethics committee at each participating center has reviewed the trial protocol and patient information as well as informed consent form and ethical approval has been granted. All women eligible for participation will be asked to give written informed consent prior to participation in the trial. Results will be published in peer-reviewed journals and disseminated at national and international conferences.

DISCUSSION

HDP are still the leading cause for maternal and perinatal morbidity and mortality worldwide and therefore constitute a major socioeconomical burden. The prevalence varies from 2-8% in the industrialized world and while the underlying pathophysiology is still largely unknown [23] an underlying cardiovascular origin of the disease is suspected. [24] Novel therapeutic strategies to prevent long-term consequences of the disease are necessary. In this observational cohort study maternal hemodynamic changes will be evaluated in women with HDP as well as in healthy control subjects to gain more insight into cardiovascular changes in women with HDP. Furthermore, the pre- and postnatal cardiovascular changes as well as effects of antihypertensive treatment will be further investigated in a preeclamptic rat model. Apart from gaining additional information on the underlying pathophysiology of preeclampsia, the effects of antihypertensive therapy on maternal hemodynamics in HDP will be investigated in women with gestational hypertension and preeclampsia. Recent studies investigating the effect of antihypertensive treatment on maternal hemodynamics in women suffering from HDP indicate that the outcome can be improved by hemodynamically guided antihypertensive therapy. [14]

For peer review only

Sufficient data to support this, however, are lacking. Therefore, the goal of this study is to prove that maternal hemodynamic assessment is beneficial in women with HDP and leads to an overall improvement of maternal and neonatal outcomes. This study will influence the development of new strategies in the management of HDP and hopefully warrant implementation of hemodynamic assessment in women with HDP.

Funding: This work is funded by an ERA-CVD Grant for Early Career Scientists through the Austrian, German and Norwegian Research fund (Austria: FWF-Grant number I 4149-B, Germany: Grant number 01KL1911, Norway: Research Council of Norway, project number 297333).

Ethical Approval: Ethical approval has been granted from each of the involved institutions.
Vienna Ethics Committee approval number 1174/2019
Berlin Ethics Committee approval number G0010/21
The Regional Committee for Medical and Health Research Ethics in South-Eastern Norway ref number 2013/2092

Competing interests: None declared.

Author contributions: JB is the general project coordinator and principal investigator of the project, MS and NH are project coordinators and principial investigators at their respective sites. JB, MS and NH conceived and designed the study. JB, MS, NH, EK, AK and PP drafted the manuscript. All authors have critically read, revised and approved the final manuscript.

For peer review only

REFERENCES

1. Mol BWJ, Roberts CT, Thangaratinam S, Magee LA, de Groot CJM, Hofmeyr GJ. Pre-eclampsia. *Lancet* 2016; **387**(10022): 999-1011.
2. Khan KS, Wojdyla D, Say L, Gülmezoglu AM, Van Look PF. WHO analysis of causes of maternal death: a systematic review. *Lancet* 2006; **367**(9516): 1066-74.
3. Eghbali M, Deva R, Alioua A, et al. Molecular and functional signature of heart hypertrophy during pregnancy. *Circ Res* 2005; **96**(11): 1208-16.
4. Lykke JA, Langhoff-Roos J, Sibai BM, Funai EF, Triche EW, Paidas MJ. Hypertensive Pregnancy Disorders and Subsequent Cardiovascular Morbidity and Type 2 Diabetes Mellitus in the Mother. *Hypertension* 2009; **53**(6): 944-51.
5. Mongraw-Chaffin ML, Cirillo PM, Cohn BA. Preeclampsia and cardiovascular disease death: prospective evidence from the child health and development studies cohort. *Hypertension* 2010; **56**(1): 166-71.
6. Melchiorre K, Sutherland GR, Liberati M, Thilaganathan B. Preeclampsia Is Associated With Persistent Postpartum Cardiovascular Impairment. *Hypertension* 2011; **58**(4): 709-15.
7. Melchiorre K, Sutherland GR, Baltabaeva A, Liberati M, Thilaganathan B. Maternal cardiac dysfunction and remodeling in women with preeclampsia at term. *Hypertension* 2011; **57**(1): 85-93.
8. Melchiorre K, Sutherland G, Sharma R, Nanni M, Thilaganathan B. Mid-gestational maternal cardiovascular profile in preterm and term pre-eclampsia: a prospective study. *Bjog* 2013; **120**(4): 496-504.
9. Melchiorre K, Sharma R, Thilaganathan B. Cardiovascular implications in preeclampsia: an overview. *Circulation* 2014; **130**(8): 703-14.
10. Vinayagam D, Gutierrez J, Binder J, Mantovani E, Thilaganathan B, Khalil A. Impaired maternal hemodynamics in morbidly obese women: a case-control study. *Ultrasound Obstet Gynecol* 2017; **50**(6): 761-5.
11. Gagliardi G, Tiralongo GM, LoPresti D, et al. Screening for pre-eclampsia in the first trimester: role of maternal hemodynamics and bioimpedance in non-obese patients. *Ultrasound in Obstetrics & Gynecology* 2017; **50**(5): 584-8.
12. McLaughlin K, Scholten RR, Kingdom JC, Floras JS, Parker JD. Should Maternal Hemodynamics Guide Antihypertensive Therapy in Preeclampsia? *Hypertension* 2018; **71**(4): 550-6.
13. Khalil A, Jauniaux E, Harrington K. Antihypertensive therapy and central hemodynamics in women with hypertensive disorders in pregnancy. *Obstet Gynecol* 2009; **113**(3): 646-54.
14. Stott D, Papastefanou I, Paraschiv D, Clark K, Kametas NA. Serial hemodynamic monitoring to guide treatment of maternal hypertension leads to reduction in severe hypertension. *Ultrasound Obstet Gynecol* 2017; **49**(1): 95-103.
15. Zeisler H, Llurba E, Chantraine F, et al. Predictive Value of the sFlt-1:PlGF Ratio in Women with Suspected Preeclampsia. *N Engl J Med* 2016; **374**(1): 13-22.
16. Sugulle M, Herse F, Hering L, Mockel M, Dechend R, Staff AC. Cardiovascular biomarker midregional proatrial natriuretic peptide during and after preeclamptic pregnancies. *Hypertension* 2012; **59**(2): 395-401.
17. Sabriá E, Lequerica-Fernández P, Lafuente-Ganuza P, et al. Addition of N-terminal pro-B natriuretic peptide to soluble fms-like tyrosine kinase-1/placental growth factor ratio > 38 improves prediction of pre-eclampsia requiring delivery within 1 week: a longitudinal cohort study. *Ultrasound Obstet Gynecol* 2018; **51**(6): 758-67.

For peer review only

18. Verlohren S, Perschel FH, Thilaganathan B, et al. Angiogenic Markers and Cardiovascular Indices in the Prediction of Hypertensive Disorders of Pregnancy. *Hypertension* 2017; **69**(6): 1192-7.
19. Stepan H, Kuse-Föhl S, Klockenbusch W, et al. Diagnosis and Treatment of Hypertensive Pregnancy Disorders. Guideline of DGGG (S1-Level, AWMF Registry No. 015/018, December 2013). *Geburtshilfe Frauenheilkd* 2015; **75**(9): 900-14.
20. Faul F, Erdfelder E, Lang AG, Buchner A. G*Power 3: a flexible statistical power analysis program for the social, behavioral, and biomedical sciences. *Behav Res Methods* 2007; **39**(2): 175-91.
21. Moe K, Sugulle M, Dechend R, Angel K, Staff AC. Functional and structural vascular biomarkers in women 1 year after a hypertensive disorder of pregnancy. *Pregnancy Hypertens* 2020; **21**: 23-9.
22. Kvehaugen AS, Dechend R, Ramstad HB, Troisi R, Fugelseth D, Staff AC. Endothelial function and circulating biomarkers are disturbed in women and children after preeclampsia. *Hypertension* 2011; **58**(1): 63-9.
23. Sibai BM. Etiology and management of postpartum hypertension-preeclampsia. *American Journal of Obstetrics and Gynecology* 2012; **206**(6): 470-5.
24. Thilaganathan B, Kalafat E. Cardiovascular System in Preeclampsia and Beyond. *Hypertension* 2019; **73**(3): 522-31.

Figure legend:

Figure 1: Hemodynamic and laboratory assessments at each visit

Figure 2: Trial algorithm: Timing of measurements within the patient cohort

AT … antihypertensive therapy

For peer review only

For peer review only

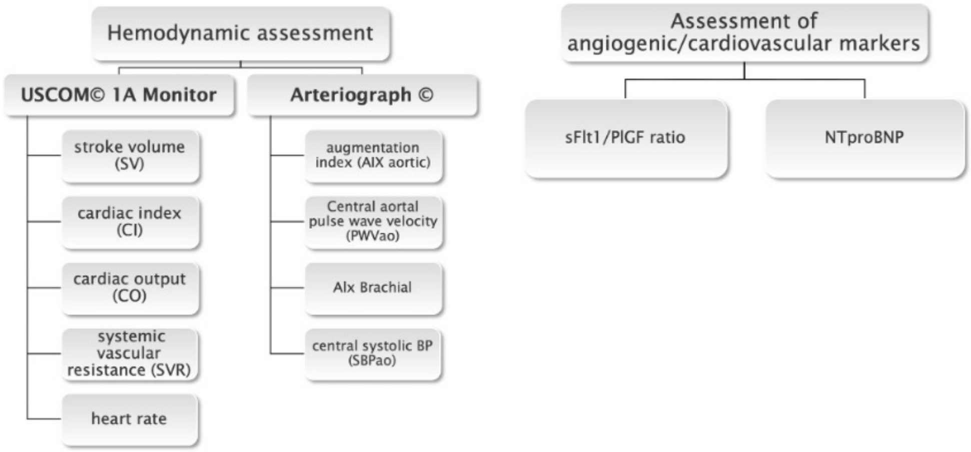

Figure 1: Hemodynamic and laboratory assessments at each visit

153x75mm (144 x 144 DPI)

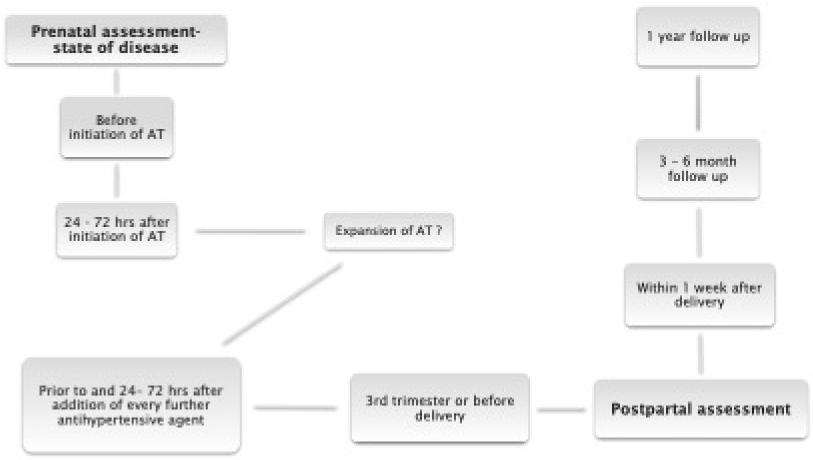

Figure 2: Trial algorithm: Timing of measurements within the patient cohort
AT … antihypertensive therapy

157x86mm (72 x 72 DPI)

SPIRIT

STANDARD PROTOCOL ITEMS: RECOMMENDATIONS FOR INTERVENTIONAL TRIALS

SPIRIT 2013 Checklist: Recommended items to address in a clinical trial protocol and related documents*

Section/item	Item No	Description
**Administrative information**		
Title	1	Descriptive title identifying the study design, population, interventions, and, if applicable, trial acronym page 1
Trial registration	2a	Trial identifier and registry name. If not yet registered, name of intended registry N/A
	2b	All items from the World Health Organization Trial Registration Data Set N/A
Protocol version	3	Date and version identifier N/A
Funding	4	Sources and types of financial, material, and other support – page 12
Roles and responsibilities	5a	Names, affiliations, and roles of protocol contributors page 12
	5b	Name and contact information for the trial sponsor page 12
	5c	Role of study sponsor and funders, if any, in study design; collection, management, analysis, and interpretation of data; writing of the report; and the decision to submit the report for publication, including whether they will have ultimate authority over any of these activities N/A
	5d	Composition, roles, and responsibilities of the coordinating centre, steering committee, endpoint adjudication committee, data management team, and other individuals or groups overseeing the trial, if applicable (see Item 21a for data monitoring committee) N/A
**Introduction**		
Background and rationale	6a	Description of research question and justification for undertaking the trial, including summary of relevant studies (published and unpublished) examining benefits and harms for each intervention page 4 + 5
	6b	Explanation for choice of comparators page 4
Objectives	7	Specific objectives or hypotheses page 4

For peer review only

Trial design	8	Description of trial design including type of trial (eg, parallel group, crossover, factorial, single group), allocation ratio, and framework (eg, superiority, equivalence, noninferiority, exploratory) page 5

Methods: Participants, interventions, and outcomes

Study setting	9	Description of study settings (eg, community clinic, academic hospital) and list of countries where data will be collected. Reference to where list of study sites can be obtained pages 5, 8, 9
Eligibility criteria	10	Inclusion and exclusion criteria for participants. If applicable, eligibility criteria for study centres and individuals who will perform the interventions (eg, surgeons, psychotherapists) page 6
Interventions	11a	Interventions for each group with sufficient detail to allow replication, including how and when they will be administered
	11b	Criteria for discontinuing or modifying allocated interventions for a given trial participant (eg, drug dose change in response to harms, participant request, or improving/worsening disease) N/A
	11c	Strategies to improve adherence to intervention protocols, and any procedures for monitoring adherence (eg, drug tablet return, laboratory tests) N/A
	11d	Relevant concomitant care and interventions that are permitted or prohibited during the trial N/A
Outcomes	12	Primary, secondary, and other outcomes, including the specific measurement variable (eg, systolic blood pressure), analysis metric (eg, change from baseline, final value, time to event), method of aggregation (eg, median, proportion), and time point for each outcome. Explanation of the clinical relevance of chosen efficacy and harm outcomes is strongly recommended pages 6-10
Participant timeline	13	Time schedule of enrolment, interventions (including any run-ins and washouts), assessments, and visits for participants. A schematic diagram is highly recommended (see Figure) pages 5-9, see Figure 2
Sample size	14	Estimated number of participants needed to achieve study objectives and how it was determined, including clinical and statistical assumptions supporting any sample size calculations page 8
Recruitment	15	Strategies for achieving adequate participant enrolment to reach target sample size page 5

Methods: Assignment of interventions (for controlled trials) N/A

Allocation:

Sequence generation	16a	Method of generating the allocation sequence (eg, computer-generated random numbers), and list of any factors for stratification. To reduce predictability of a random sequence, details of any planned restriction (eg, blocking) should be provided in a separate document that is unavailable to those who enrol participants or assign interventions
Allocation concealment mechanism	16b	Mechanism of implementing the allocation sequence (eg, central telephone; sequentially numbered, opaque, sealed envelopes), describing any steps to conceal the sequence until interventions are assigned
Implementation	16c	Who will generate the allocation sequence, who will enrol participants, and who will assign participants to interventions
Blinding (masking)	17a	Who will be blinded after assignment to interventions (eg, trial participants, care providers, outcome assessors, data analysts), and how
	17b	If blinded, circumstances under which unblinding is permissible, and procedure for revealing a participant's allocated intervention during the trial
**Methods: Data collection, management, and analysis**		
Data collection methods	18a	Plans for assessment and collection of outcome, baseline, and other trial data, including any related processes to promote data quality (eg, duplicate measurements, training of assessors) and a description of study instruments (eg, questionnaires, laboratory tests) along with their reliability and validity, if known. Reference to where data collection forms can be found, if not in the protocol page 11
	18b	Plans to promote participant retention and complete follow-up, including list of any outcome data to be collected for participants who discontinue or deviate from intervention protocols N/A
Data management	19	Plans for data entry, coding, security, and storage, including any related processes to promote data quality (eg, double data entry; range checks for data values). Reference to where details of data management procedures can be found, if not in the protocol page 11
Statistical methods	20a	Statistical methods for analysing primary and secondary outcomes. Reference to where other details of the statistical analysis plan can be found, if not in the protocol page 10
	20b	Methods for any additional analyses (eg, subgroup and adjusted analyses) page 10
	20c	Definition of analysis population relating to protocol non-adherence (eg, as randomised analysis), and any statistical methods to handle missing data (eg, multiple imputation) page 10

For peer review only - http://bmjopen.bmj.com/site/about/guidelines.xhtml

Methods: Monitoring

Data monitoring	21a	Composition of data monitoring committee (DMC); summary of its role and reporting structure; statement of whether it is independent from the sponsor and competing interests; and reference to where further details about its charter can be found, if not in the protocol. Alternatively, an explanation of why a DMC is not needed DMC not needed as this is a prospective cohort study, not an intervention study.
	21b	Description of any interim analyses and stopping guidelines, including who will have access to these interim results and make the final decision to terminate the trial N/A
Harms	22	Plans for collecting, assessing, reporting, and managing solicited and spontaneously reported adverse events and other unintended effects of trial interventions or trial conduct N/A
Auditing	23	Frequency and procedures for auditing trial conduct, if any, and whether the process will be independent from investigators and the sponsor N/A

Ethics and dissemination page 11

Research ethics approval	24	Plans for seeking research ethics committee/institutional review board (REC/IRB) approval Ethics approval already received, see page 11
Protocol amendments	25	Plans for communicating important protocol modifications (eg, changes to eligibility criteria, outcomes, analyses) to relevant parties (eg, investigators, REC/IRBs, trial participants, trial registries, journals, regulators) N/A
Consent or assent	26a	Who will obtain informed consent or assent from potential trial participants or authorised surrogates, and how (see Item 32) page 5
	26b	Additional consent provisions for collection and use of participant data and biological specimens in ancillary studies, if applicable N/A
Confidentiality	27	How personal information about potential and enrolled participants will be collected, shared, and maintained in order to protect confidentiality before, during, and after the trial page 11
Declaration of interests	28	Financial and other competing interests for principal investigators for the overall trial and each study site page 12
Access to data	29	Statement of who will have access to the final trial dataset, and disclosure of contractual agreements that limit such access for investigators page 10-11
Ancillary and post-trial care	30	Provisions, if any, for ancillary and post-trial care, and for compensation to those who suffer harm from trial participation N/A

For peer review only - http://bmjopen.bmj.com/site/about/guidelines.xhtml

Dissemination policy	31a	Plans for investigators and sponsor to communicate trial results to participants, healthcare professionals, the public, and other relevant groups (eg, via publication, reporting in results databases, or other data sharing arrangements), including any publication restrictions page 11
	31b	Authorship eligibility guidelines and any intended use of professional writers n/A
	31c	Plans, if any, for granting public access to the full protocol, participant-level dataset, and statistical code N/A
**Appendices**		
Informed consent materials	32	Model consent form and other related documentation given to participants and authorised surrogates can be added as appendix if required
Biological specimens	33	Plans for collection, laboratory evaluation, and storage of biological specimens for genetic or molecular analysis in the current trial and for future use in ancillary studies, if applicable N/A

*It is strongly recommended that this checklist be read in conjunction with the SPIRIT 2013 Explanation & Elaboration for important clarification on the items. Amendments to the protocol should be tracked and dated. The SPIRIT checklist is copyrighted by the SPIRIT Group under the Creative Commons "Attribution-NonCommercial-NoDerivs 3.0 Unported" license.

For peer review only

Maternal hemodynamics in Hypertensive Disorders of Pregnancy under antihypertensive therapy (HyperDiP): study protocol for a prospective observational case control study

Journal:	*BMJ Open*
Manuscript ID	bmjopen-2022-065444.R1
Article Type:	Protocol
Date Submitted by the Author:	23-Feb-2023

Complete List of Authors:	Palmrich, Pilar; Hospital of the Medical University of Vienna Division of Obstetrics and Feto-Maternal Medicine<br>Haase, Nadine; Experimental and Clinical Research Center, a cooperation between the Max Delbrück Center for Molecular Medicine in the Helmholtz Association and Charité Universitätsmedizin ; Max - Delbrück Center for Molecular Medicine in the Helmholtz Association (MDC<br>Sugulle, Meryam ; Oslo University Hospital, Division of Gynecology and Obstetrics; University of Oslo Faculty of Medicine<br>Kalafat, E. ; Koc University Hospital, Department of Obstetrics and Gynecology,<br>Khalil, Asma; St George's Hospital, Fetal Medicine Unit<br>Binder, Julia; Hospital of the Medical University of Vienna Division of Obstetrics and Feto-Maternal Medicine

**Primary Subject Heading**:	Obstetrics and gynaecology
Secondary Subject Heading:	Cardiovascular medicine
Keywords:	Maternal medicine < OBSTETRICS, Ultrasonography, Hypertension < CARDIOLOGY

SCHOLARONE™
Manuscripts

Maternal hemodynamics in Hypertensive Disorders of Pregnancy under antihypertensive therapy (HyperDiP): study protocol for a prospective observational case control study

Pilar Palmrich[1], Nadine Haase[2-5], Meryam Sugulle[6-7], Erkan Kalafat [8], Asma Khalil[9], Julia Binder[1]

[1] Department of Obstetrics and Feto-Maternal Medicine, Medical University of Vienna
[2] Experimental and Clinical Research Center, a cooperation between the Max Delbrück Center for Molecular Medicine in the Helmholtz Association and Charité Universitätsmedizin Berlin, Germany
[3] Max - Delbrück Center for Molecular Medicine in the Helmholtz Association (MDC), Berlin, Germany
[4] Charité – Universitätsmedizin Berlin, corporate member of Freie Universität Berlin, Humboldt-Universität zu Berlin, Experimental and Clinical Research Center, Berlin, Germany
[5] DZHK (German Centre for Cardiovascular Research), partner site Berlin, Germany
[6] Division of Gynecology and Obstetrics, Oslo University Hospital, Ullevål, Oslo, Norway
[7] University of Oslo, Faculty of Medicine, Oslo, Norway
[8] Department of Obstetrics and Gynecology, Koc University Hospital, Istanbul, Turkey
[9] Fetal Medicine Unit, St. George's Hospital St George's University of London, London, United Kingdom

Corresponding author:

Priv.-Doz. Dr.med.univ. Julia Binder, PhD

Department of Obstetrics and Gynecology, Division of Obstetrics and feto-maternal Medicine, Medical University of Vienna, Vienna, Austria

Email: julia.binder@meduniwien.ac.at

Phone: +43 (0)1 40400 – 28210

For peer review only

ABSTRACT:

Introduction: Hypertensive disorders of pregnancy (HDP) are associated with a high incidence of maternal and perinatal morbidity and mortality. HDP, in particular preeclampsia, have been determined as risk factors for future cardiovascular disease (CVD). Recently, the common hypothesis of preeclampsia being a placental disorder was challenged as numerous studies show evidence for short- and long-term cardiovascular changes in pregnancies affected by HDP, suggesting a cardiovascular origin of the disease. Despite new insights into the pathophysiology of HDP, concepts of therapy remain unchanged and evidence for improved maternal and neonatal outcome by using antihypertensive agents is lacking.

Methods and Analysis: A prospective observational case control study, including 100 women with HDP and 100 healthy controls, which will assess maternal hemodynamics using the USCOM©1A Monitor and Arteriograph© along with cardiovascular markers (sFlt-1/PlGF, NTproBNP) in women with HDP under antihypertensive therapy, including a follow-up at three months and one-year post-partum, will be conducted over a 50-month period in Vienna. A prospective, longitudinal study of cardiovascular surrogate markers conducted in Oslo will serve as a comparative cohort for the Vienna cohort of hemodynamic parameters in pregnancy including a longer follow up period of up to 3 years post-partum. Each site will provide a dataset of a patient group and a control group and will be assessed for the outcome categories USCOM©1A measurements; Arteriograph© measurements; Angiogenic marker measurements. To estimate the effect of antihypertensive therapy on outcome parameters, odds ratios with 95% confidence intervals will be computed. Longitudinal changes of outcome parameters will be compared between normotensive and hypertensive pregnancies using mixed effects models.

Ethics and Dissemination: Ethical approval has been granted to all participating centers. Results will be published in international peer-reviewed journals and will be presented at national and international conferences.

For peer review only

STRENGTHS AND LIMITATIONS

- A strength of this study is that changes in maternal hemodynamics in combination with cardiovascular markers will be assessed prior to and after antihypertensive treatment in women with hypertensive disorders of pregnancy compared to a healthy gestational age matched control group.
- Another strength of the analysis is the comparison between single and combined oral antihypertensives.
- A potential limitation of the study worth noting is that the comparison between different groups of antihypertensive agents might pose a challenge as the sample sizes within the respective groups will be small as we assume that a large proportion of patients will receive only one antihypertensive drug.

For peer review only

BACKGROUND

Hypertensive disorders of pregnancy (HDP) are still the leading cause of maternal and perinatal morbidity and mortality worldwide. [1,2] Women undergo significant physiological systemic changes during pregnancy. Regarding the cardiovascular system, pregnancy acts as a cardiac stress model. Despite echocardiographically detectable changes, most pregnant women tolerate the inflicted cardiac stress without development of clinical symptoms. [3] HDP, in particular the pregnancy specific syndrome preeclampsia, have been found to be common risk factors for future cardiovascular disease (CVD), even in previously healthy women without evidence of previous cardiovascular disease. [4,5] The underlying pathophysiology and pathomechanism leading to preeclampsia are not yet fully understood and have been the focus of research in the last decades. Recently, the common hypothesis of preeclampsia being a placental disorder was challenged as a number of studies show evidence for short and long term cardiovascular changes in pregnancies affected by preeclampsia, possibly suggesting a cardiovascular origin of the disease. These cardiovascular changes include impaired cardiac output, an increase in systemic vascular resistance and left ventricular diastolic dysfunction especially in women affected by early onset preeclampsia. [6-10] Studies on maternal hemodynamic changes in women with preeclampsia revealed persistent long-term cardiac alterations of up to two years after preeclampsia and an increased lifetime risk of essential hypertension and cardiovascular disease. [11] Although there are new insights into a potential cardiovascular origin of the disease, the strategies of treating preeclampsia have not changed fundamentally in the last years. Furthermore, there is no international consensus on when and how increased blood pressure in pregnancy should be treated. [12] Studies of maternal hemodynamics in women suffering from HDP on antihypertensive treatment indicate effectiveness of hemodynamically guided antihypertensive therapy. [13] The data to support this, however, are limited. Khalil et al. demonstrated significantly lower arterial stiffness in women treated with alpha-methyldopa, the first line treatment of hypertension in pregnancy [14] in many countries. A study by Stott et al., evaluating antihypertensive therapy with labetalol and nifedipine guided by hemodynamic parameters in pregnant women with HDP, was able to demonstrate a significant decrease in episodes of severe hypertension when treatment was guided by hemodynamic parameters. [15] Studies on non-pregnant women undergoing hemodynamic monitoring while receiving antihypertensive therapy support this approach of a hemodynamically guided therapy when blood pressure is resistant or refractory. [12] Furthermore, a recent study by Mulder et al., assessing possible risk reduction of recurrent preeclampsia by introducing early tailored treatment of nonphysiological hemodynamic changes during pregnancy in women with previous preeclampsia, showed a significant risk reduction of recurrent preeclampsia in this high risk population.[16] Cardiovascular markers such as soluble fms like kinase 1 (sFlt-1) and placental like growth factor (PIGF) have been

For peer review only

introduced into clinical practice to help estimating the time to delivery in women with preeclampsia. [17] A study by Sugulle et al. indicated the usefulness of midregional proatrial natriuretic peptide (MR-proANP) as a biomarker in preeclampsia, both in humans and in a rat model, likely to reflect cardiovascular hemodynamic stress in these women.[18] N-terminal pro-B type natriuretic peptide (NT-pro BNP) is known to be released by cardiomyocytes due to ischemia and increased mechanical strain seen in women with preeclampsia but also in non-pregnant patients with heart failure, showed potential in predicting time to delivery in combination with the sFlt1/PlGF ratio within one week. [19] In a prospective study assessing biophysical and biochemical markers of cardiovascular strain and placental dysfunction in patients with HDP and healthy controls, Verlohren et al. also demonstrated the additive predictive value of total peripheral resistance index (TPRI), with additional moderate contribution of NT-proBNP and cardiac index (CI) in combination with sFlt-1/PlGF ratio for the prediction of HDP. [20]

For peer review only

OBJECTIVES:

This project aims to evaluate cardiovascular parameters in women affected by HDP by assessing maternal hemodynamic function during pregnancy as well as in the postpartum period up until 3 years after delivery. The focus of this study is to evaluate maternal hemodynamics as well as angiogenic and cardiovascular markers (sFlt-1/PlGF, NT-pro BNP) under antihypertensive therapy in women affected by HDP compared to a healthy control group matched for gestational age, in order to assess whether cardiac indices should be used as a guiding tool for antihypertensive therapy in these women. In this study, we focus on changes in maternal hemodynamics secondary to antihypertensive treatment, also comparing single and combined oral antihypertensive therapy in women with HDP.

METHODS

Assessment of maternal hemodynamic function in pregnancy and postpartum - Vienna:

This is a prospective case control study over a 50-month period, assessing women with hypertensive disorders of pregnancy (HDP), including both gestational hypertension and preeclampsia, as well as a control group composed of healthy pregnant women matched for gestational age. All women will be recruited at the high-risk maternal outpatient clinic and antenatal ward at the Department of Obstetrics and fetomaternal Medicine at the Medical University of Vienna. HDP, including gestational hypertension and preeclampsia, will be diagnosed according to the International Society for Hypertensive Disorders of Pregnancy (ISSHP) 2021 revised criteria. [21] Gestational hypertension is defined as de-novo systolic blood

For peer review only - http://bmjopen.bmj.com/site/about/guidelines.xhtml

pressure of ≥140 mm Hg and/or diastolic blood pressure ≥90 mm Hg on 2 separate occasions ≥24 hours apart after 20 weeks of gestation. Preeclampsia is defined as de novo hypertension (≥140/90 mmHg) after 20 weeks of gestation with the coexistence of proteinuria (protein/creatinine ratio ≥30 mg/mmol), other maternal organ dysfunction including hepatic dysfunction (elevated transaminases, alanine aminotransferase or aspartate aminotransferase >40 IU/L) with or without right upper quadrant or epigastric pain, renal insufficiency (creatinine ≥100 micromol/L antenatally, ≥130 postnatally), neurological symptoms (e.g. altered mental state, severe headaches, persistent visual scotoma, eclampsia), hematological complications (thrombocytopenia - platelet count <150 000/µL, disseminated intravascular coagulation, hemolysis), pulmonary edema or uteroplacental dysfunction (fetal growth restriction). [21]

Women fulfilling eligibility criteria (see Box 1 and 2) will undergo non-invasive assessment of maternal hemodynamics including determination of heart rate, cardiac output, cardiac index, stroke volume and systemic vascular resistance using USCOM© 1A Monitor, non-invasive assessment of arterial stiffness using Arteriograph® as well as measurement of sFlt1/PlGF ratio and NTproBNP by taking blood samples at each hemodynamic assessment (figure 1).

Box 1: Inclusion criteria

Pregnant women 20+0 and 42+0 weeks of gestation with a

- singleton pregnancy
- Aged 18 years or older
- Able to give valid informed consent
- Presenting with gestational hypertension or preeclampsia

Box 2: Exclusion criteria

- Major fetal anomaly or aneuploidy/genetic syndrome
- Maternal and fetal cardiac defects (e.g. congenital and non-congenital cardiac anomalies, heart failure, coronary heart disease)
- Systemic cardiovascular diseases (e.g. systemic lupus erythematodes with end organ dysfunction)
- Diabetes mellitus type I and II
- Chronic hypertension (predating pregnancy or onset < 20 weeks of gestation)
- Chronic kidney disease (e.g. glomerulonephritis, polycystic kidney disease, renal insufficiency predating pregnancy)

For peer review only - http://bmjopen.bmj.com/site/about/guidelines.xhtml

Primary and secondary outcome measures are summarized in Box 3 to 5.

Box 3: Primary Outcomes

- **Comparison of maternal hemodynamics (figure 1) and angiogenic factors (sFlt-1/PIGF, NTproBNP) in women with HDP compared to healthy controls**
- **Assessment of maternal hemodynamics (figure 1) in women with hypertensive disorders of pregnancy under antihypertensive therapy:**
  - Assessment of hemodynamic parameters before and after treatment
  - Assessment of maternal hemodynamic parameters between women treated with different groups of antihypertensive agents
- **Assessment of angiogenic factors in women with HDP under antihypertensive therapy:**
  - Correlation of changes in hemodynamic parameters and angiogenic factors in women under antihypertensive treatment

Box 4: Secondary Outcomes: maternal adverse outcome

- Hepatic dysfunction (elevated transaminases, alanine aminotransferase or aspartate aminotransferase >40 IU/L)
- Acute renal insufficiency (defined as creatinine ≥100 micromol/L antenatally, or ≥130 postnatally) or need for dialysis
- Placental abruption (Retroplacental clot or associated with preterm delivery or fetal demise) described clinically
- Admission to the intensive care unit
- Days of hospital admission
- Mode of delivery
- Post-partum hemorrhage (defined as more than >500 ml blood loss after delivery
- Development of chronic hypertension in the follow up period

Box 5: Secondary Outcomes: perinatal/neonatal outcome

- Gestational age at delivery
- Birth weight

For peer review only - http://bmjopen.bmj.com/site/about/guidelines.xhtml

- Stillbirth
- Neonatal death
- Neonatal morbidity (admission to neonatal unit for more than 48 hours, respiratory distress syndrome, need for intubation, intraventricular hemorrhage, confirmed infection, necrotizing enterocolitis, seizures, encephalopathy, retinopathy of prematurity)
- Need for admission to Neonatal Intensive Care Unit (NICU)
- Need for admission to Special Care Baby Unit
- Number of bed nights in NICU associated with delivery for PE

USCOM© 1A Monitor is a device using continuous wave Doppler through a non-imaging probe in the suprasternal notch. It assesses velocity time integrals of transaortic blood flow at the left ventricular outflow tract, obtaining a complete hemodynamic profile. The Arteriograph® device is a validated method of non-invasive assessment of arterial stiffness by measuring pulse wave velocity (PWVao), aortic augmentation index (Aix) and central systolic blood pressure (SBPao) using a simple upper arm cuff. Serum sFlt1/PIGF ratio and NT- proBNP will be measured using an automated immune analyzer (Roche Diagnostics, Germany) at each hemodynamic assessment. Initial evaluation, if possible, will take place prior to starting antihypertensive treatment, followed by assessment after 24-72 hours after initiation of antihypertensive therapy. The same measurements will be repeated if therapy is extended by another antihypertensive agent. If no additional antihypertensive therapy is necessary, another assessment will be performed in the third trimester or prior to delivery if indicated due to critical maternal or neonatal situation. In the postpartum period assessments will be undertaken within one week after delivery, and after a 3 to 6 month and a one year follow up period (see figure 2). Antihypertensive treatment will be initiated as per local protocol, which is based on the updated AWMF (Association of the scientific medical societies in Germany) guideline. [22] The control group will have serial hemodynamic assessment and blood sampling for cardiovascular parameters (as described for the cases group previously) in a four-week interval during pregnancy and within a one-week interval after birth, after 3 to 6 months and one year post-partum.

For peer review only

Sample size

The sample size estimation was done using the G*Power software (version 3.1.9.6, Universität Düsseldorf, Germany). [23] We estimated the sample size assuming a repeated measures ANCOVA model to test the between factor significance with the following parameters; effect size f: 0.15; error probability: 0.05, power: 0.90. We used a small to moderate generic effect size instead of tailoring it to a specific parameter, as a number of cardiovascular parameters will be compared between the groups. We further assumed an average of four measurements per group with a weak correlation between repeated measures (0.2). Under these

For peer review only - http://bmjopen.bmj.com/site/about/guidelines.xhtml

assumptions, a total sample size of 220 (110 patients in each) was estimated to reach the targeted power level.

Postnatal longitudinal assessment of cardiovascular surrogate markers - Oslo:

Oslo University Hospital, Ullevål will provide a comparative cohort for the Vienna cohort of hemodynamics in pregnancy. The Norwegian "HAPPY" - "Health after pregnancy complications" study, which currently comprises 221 women at 1-year and 108 women at 3 year follow up, among others including women after HDP and control patients, will be able to provide longitudinal data on cardiovascular surrogate markers such as endothelial function, carotid artery wall structure and aortic stiffness. Also, circulating biomarkers for CVD risk, such as systemic inflammatory marker hsCRP, and systemic "metabolic stress" markers leptin, insulin resistance (HoMa score), fasting blood lipids (Triglycerides, LDL-Cholesterol, HDL-C, oxLDL), measured by immunoassays and multiplex assays. [24] Additionally, the Oslo Pregnancy Biobank, contains maternal blood and umbilical blood as well as placental tissue samples for assessment of morphological changes and will add additional comparative data. Furthermore, the CHASE study "Cardiovascular health after pregnancy complications" will serve as an another postpartum cohort, including blood samples and surrogate markers for endothelial function of 40 mother-child pairs 5 to 8 years postpartum. [25]

Timeline:

The trial commenced in July 2019 and the planned end-date is September 2023.

Patient and Public Involvement:

Patients or the public will not be involved in the design, conduct, reporting, or dissemination plans of our research.

STATISTICAL ANALYSIS

Each site will provide a unique dataset comprising a patient group and a control group. The patient group will be divided into the two subgroups early onset preeclampsia (onset before 34 weeks of gestation) and late onset preeclampsia (onset after 34 weeks of gestation) for statistical analysis. Three sets of analyses are planned for each outcome category (USCOM measurements; Arteriograph measurements; Angiogenic marker measurements).

Baseline differences between hypertensive and normotensive pregnancies

For peer review only - http://bmjopen.bmj.com/site/about/guidelines.xhtml

Measurements at the time of enrolment will be used for this analysis. Variables will be described using location and scale parameters (mean, standard deviation, median, interquartile range etc.) appropriate for their distribution characteristics. Groups will be compared after matching for predefined confounders (BMI and gestational age at assessment). Mean differences or odds ratio with 95% confidence intervals will be reported to describe the effect sizes. Effect sizes will be estimated with generalized estimating equations using match identifiers as a cluster.

Estimating the effect of antihypertensive therapy on outcome parameters

Measurements at the time of enrollment and after the initiation of antihypertensive therapy (24 to 72 hours) will be used for this analysis. Paired measurements will be used to estimate the mean differences or odds ratio with 95% confidence intervals. For variables with skewed distributions, median differences and nonparametric bootstrapped confidence intervals will be reported. Also, clinical factors associated with before and after changes will be investigated and will be adjusted if necessary.

Longitudinal change of outcome parameters between hypertensive and normotensive pregnancies

Repeat measurements from normotensive pregnancies and hypertensive pregnancies which were not medicated will be used for this analysis. A two-stage approach will be adopted for analysis. First, generalized linear mixed-effects models (GLMM) with appropriate link function and random slopes for patient identifiers will be used to model the change of outcome variables with gestational age. Random effects from GLMM models will be extracted and the effect of hypertension status will be tested with linear models. All analyses will be conducted using R for Statistical Computing Software (R foundation, Vienna, Austria) performed by an appointed statistician.

DATA MONITORING

Study participants will be anonymized by assigning a sequential patient number. All data will be entered, processed and stored anonymously in a case report form the online data management program Clincase © by the study investigators.

ETHICS and DISSEMINATION

For peer review only - http://bmjopen.bmj.com/site/about/guidelines.xhtml

This study will be conducted in accordance with the principles of Good Clinical Practice (GCP). The local ethics committee at each participating center has reviewed the trial protocol and patient information as well as informed consent form and ethical approval has been granted. All women eligible for participation will be asked to give written informed consent prior to participation in the trial. Results will be published in peer-reviewed journals and disseminated at national and international conferences.

DISCUSSION

HDP are still the leading cause for maternal and perinatal morbidity and mortality worldwide and therefore constitute a major socioeconomical burden. The prevalence varies from 2-8% in the industrialized world and while the underlying pathophysiology is still largely unknown [26] an underlying cardiovascular origin of the disease is suspected. [27] Novel therapeutic strategies to prevent long-term consequences of the disease are necessary. In this observational cohort study maternal hemodynamic changes will be evaluated in women with HDP as well as in healthy control subjects to gain more insight into cardiovascular changes in women with HDP. Apart from gaining additional information on the underlying pathophysiology of preeclampsia, the effects of antihypertensive therapy on maternal hemodynamics in HDP will be investigated in women with gestational hypertension and preeclampsia. Recent studies investigating the effect of antihypertensive treatment on maternal hemodynamics in women suffering from HDP indicate that the outcome can be improved by hemodynamically guided antihypertensive therapy. [15] Sufficient data to support this, however, are lacking. Therefore, the goal of this study is to prove that maternal hemodynamic assessment is beneficial in women with HDP and leads to an overall improvement of maternal and neonatal outcomes. This study will influence the development of new strategies in the management of HDP and hopefully warrant implementation of hemodynamic assessment in women with HDP.

Funding: This work is funded by an ERA-CVD Grant for Early Career Scientists through the Austrian, German and Norwegian Research fund (Austria: FWF-Grant number I 4149-B, Germany: Grant number 01KL1911, Norway: Research Council of Norway, project number 297333).

Ethical Approval: Ethical approval has been granted from each of the involved institutions.
Ethics Committee of the Medical University of Vienna, approval number 1174/2019
Ethics Committee of the Charité, Univeritätsmedizin Berlin, approval number G0010/21
The Regional Committee for Medical and Health Research Ethics in South-Eastern Norway, approval number 2013/2092

For peer review only - http://bmjopen.bmj.com/site/about/guidelines.xhtml

Competing interests: None declared.

Author contributions: JB is the general project coordinator and principal investigator of the project, MS and NH are project coordinators and principial investigators at their respective sites. JB, MS, NH, EK, AK and PP conceived and designed the study. JB, MS, NH, EK, AK and PP drafted the manuscript. All authors have critically read, revised and approved the final manuscript.

For peer review only

REFERENCES

1. Mol BWJ, Roberts CT, Thangaratinam S, Magee LA, de Groot CJM, Hofmeyr GJ. Pre-eclampsia. *Lancet* 2016; **387**(10022): 999-1011.
2. Khan KS, Wojdyla D, Say L, Gülmezoglu AM, Van Look PF. WHO analysis of causes of maternal death: a systematic review. *Lancet* 2006; **367**(9516): 1066-74.
3. Eghbali M, Deva R, Alioua A, et al. Molecular and functional signature of heart hypertrophy during pregnancy. *Circ Res* 2005; **96**(11): 1208-16.
4. Lykke JA, Langhoff-Roos J, Sibai BM, Funai EF, Triche EW, Paidas MJ. Hypertensive Pregnancy Disorders and Subsequent Cardiovascular Morbidity and Type 2 Diabetes Mellitus in the Mother. *Hypertension* 2009; **53**(6): 944-51.
5. Mongraw-Chaffin ML, Cirillo PM, Cohn BA. Preeclampsia and cardiovascular disease death: prospective evidence from the child health and development studies cohort. *Hypertension* 2010; **56**(1): 166-71.
6. Melchiorre K, Sutherland GR, Liberati M, Thilaganathan B. Preeclampsia Is Associated With Persistent Postpartum Cardiovascular Impairment. *Hypertension* 2011; **58**(4): 709-15.
7. Melchiorre K, Sutherland GR, Baltabaeva A, Liberati M, Thilaganathan B. Maternal cardiac dysfunction and remodeling in women with preeclampsia at term. *Hypertension* 2011; **57**(1): 85-93.
8. Melchiorre K, Sutherland G, Sharma R, Nanni M, Thilaganathan B. Mid-gestational maternal cardiovascular profile in preterm and term pre-eclampsia: a prospective study. *Bjog* 2013; **120**(4): 496-504.
9. Melchiorre K, Sharma R, Thilaganathan B. Cardiovascular implications in preeclampsia: an overview. *Circulation* 2014; **130**(8): 703-14.
10. Vinayagam D, Gutierrez J, Binder J, Mantovani E, Thilaganathan B, Khalil A. Impaired maternal hemodynamics in morbidly obese women: a case-control study. *Ultrasound Obstet Gynecol* 2017; **50**(6): 761-5.
11. Gagliardi G, Tiralongo GM, LoPresti D, et al. Screening for pre-eclampsia in the first trimester: role of maternal hemodynamics and bioimpedance in non-obese patients. *Ultrasound in Obstetrics & Gynecology* 2017; **50**(5): 584-8.
12. McLaughlin K, Scholten RR, Kingdom JC, Floras JS, Parker JD. Should Maternal Hemodynamics Guide Antihypertensive Therapy in Preeclampsia? *Hypertension* 2018; **71**(4): 550-6.
13. Cottrell J, Cummings K, Jude D, Chaffin D. The effect of impedance cardiography directed antihypertensive therapy on fetal growth restriction rates and perinatal mortality in women with chronic hypertension. *Pregnancy Hypertens* 2022; **28**: 123-7.

For peer review only - http://bmjopen.bmj.com/site/about/guidelines.xhtml

14. Khalil A, Jauniaux E, Harrington K. Antihypertensive therapy and central hemodynamics in women with hypertensive disorders in pregnancy. *Obstet Gynecol* 2009; **113**(3): 646-54.

15. Stott D, Papastefanou I, Paraschiv D, Clark K, Kametas NA. Serial hemodynamic monitoring to guide treatment of maternal hypertension leads to reduction in severe hypertension. *Ultrasound Obstet Gynecol* 2017; **49**(1): 95-103.

16. Mulder EG, Ghossein-Doha C, Cauffman E, et al. Preventing Recurrent Preeclampsia by Tailored Treatment of Nonphysiologic Hemodynamic Adjustments to Pregnancy. *Hypertension* 2021; **77**(6): 2045-53.

17. Zeisler H, Llurba E, Chantraine F, et al. Predictive Value of the sFlt-1:PlGF Ratio in Women with Suspected Preeclampsia. *N Engl J Med* 2016; **374**(1): 13-22.

18. Sugulle M, Herse F, Hering L, Mockel M, Dechend R, Staff AC. Cardiovascular biomarker midregional proatrial natriuretic peptide during and after preeclamptic pregnancies. *Hypertension* 2012; **59**(2): 395-401.

19. Sabriá E, Lequerica-Fernández P, Lafuente-Ganuza P, et al. Addition of N-terminal pro-B natriuretic peptide to soluble fms-like tyrosine kinase-1/placental growth factor ratio > 38 improves prediction of pre-eclampsia requiring delivery within 1 week: a longitudinal cohort study. *Ultrasound Obstet Gynecol* 2018; **51**(6): 758-67.

20. Verlohren S, Perschel FH, Thilaganathan B, et al. Angiogenic Markers and Cardiovascular Indices in the Prediction of Hypertensive Disorders of Pregnancy. *Hypertension* 2017; **69**(6): 1192-7.

21. Magee LA, Brown MA, Hall DR, et al. The 2021 International Society for the Study of Hypertension in Pregnancy classification, diagnosis & management recommendations for international practice. *Pregnancy Hypertens* 2022; **27**: 148-69.

22. Stepan H, Kuse-Föhl S, Klockenbusch W, et al. Diagnosis and Treatment of Hypertensive Pregnancy Disorders. Guideline of DGGG (S1-Level, AWMF Registry No. 015/018, December 2013). *Geburtshilfe Frauenheilkd* 2015; **75**(9): 900-14.

23. Faul F, Erdfelder E, Lang AG, Buchner A. G*Power 3: a flexible statistical power analysis program for the social, behavioral, and biomedical sciences. *Behav Res Methods* 2007; **39**(2): 175-91.

24. Moe K, Sugulle M, Dechend R, Angel K, Staff AC. Functional and structural vascular biomarkers in women 1 year after a hypertensive disorder of pregnancy. *Pregnancy Hypertens* 2020; **21**: 23-9.

25. Kvehaugen AS, Dechend R, Ramstad HB, Troisi R, Fugelseth D, Staff AC. Endothelial function and circulating biomarkers are disturbed in women and children after preeclampsia. *Hypertension* 2011; **58**(1): 63-9.

For peer review only

26. Sibai BM. Etiology and management of postpartum hypertension-preeclampsia. *American Journal of Obstetrics and Gynecology* 2012; **206**(6): 470-5.

27. Thilaganathan B, Kalafat E. Cardiovascular System in Preeclampsia and Beyond. *Hypertension* 2019; **73**(3): 522-31.

Figure legend:

Figure 1: Hemodynamic and laboratory assessments at each visit

Figure 2: Trial algorithm: Timing of measurements within the patient cohort

AT … antihypertensive therapy

For peer review only

For peer review only - http://bmjopen.bmj.com/site/about/guidelines.xhtml

For peer review only

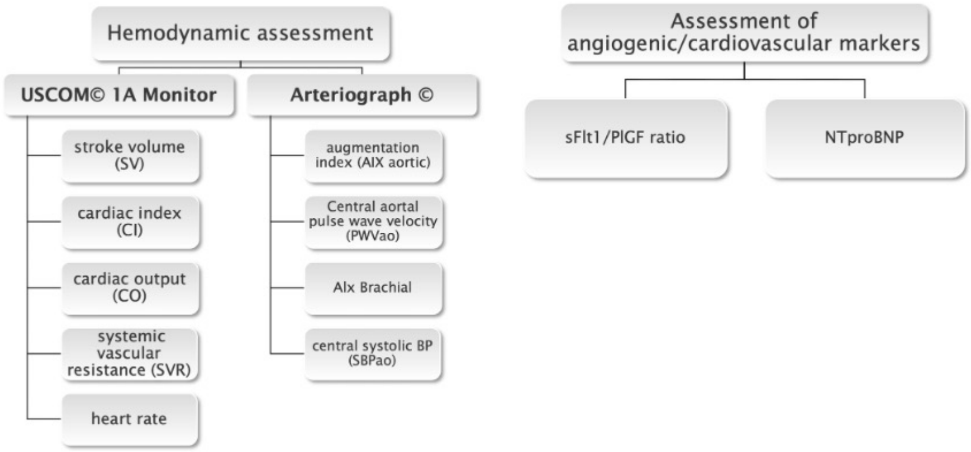

Figure 1: Hemodynamic and laboratory assessments at each visit

153x75mm (144 x 144 DPI)

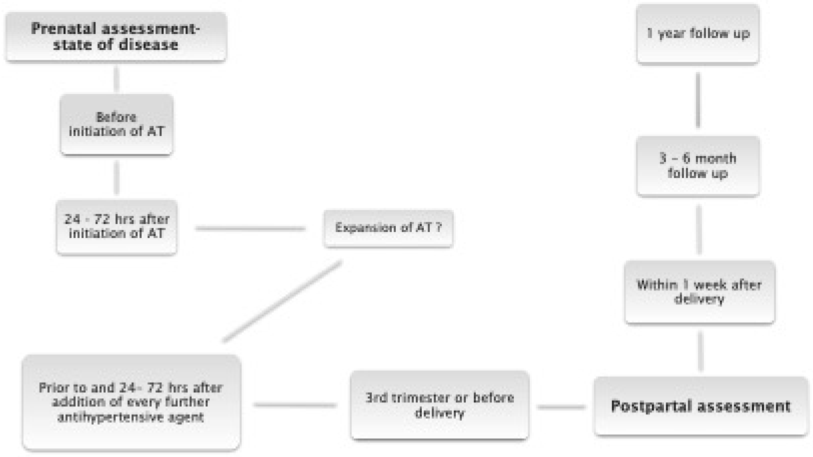

Figure 2: Trial algorithm: Timing of measurements within the patient cohort
AT … antihypertensive therapy

157x86mm (72 x 72 DPI)

STROBE Statement—Checklist of items that should be included in reports of ***case-control studies***

	Item No	Recommendation
**Title and abstract**	1	(*a*) Indicate the study's design with a commonly used term in the title or the abstract - page 1
		(*b*) Provide in the abstract an informative and balanced summary of what was done and what was found – page 2
**Introduction**		
Background/rationale	2	Explain the scientific background and rationale for the investigation being reported – pages 4-5
Objectives	3	State specific objectives, including any prespecified hypotheses – page 5
**Methods**		
Study design	4	Present key elements of study design early in the paper – page 5
Setting	5	Describe the setting, locations, and relevant dates, including periods of recruitment, exposure, follow-up, and data collection – pages 5-9
Participants	6	(*a*) Give the eligibility criteria, and the sources and methods of case ascertainment and control selection. Give the rationale for the choice of cases and controls – page 6
		(*b*) For matched studies, give matching criteria and the number of controls per case – page 5, page 9
Variables	7	Clearly define all outcomes, exposures, predictors, potential confounders, and effect modifiers. Give diagnostic criteria, if applicable – pages 5-9
Data sources/ measurement	8*	For each variable of interest, give sources of data and details of methods of assessment (measurement). Describe comparability of assessment methods if there is more than one group – page 10- 11
Bias	9	Describe any efforts to address potential sources of bias - page 8/9
Study size	10	Explain how the study size was arrived at – page 8-9
Quantitative variables	11	Explain how quantitative variables were handled in the analyses. If applicable, describe which groupings were chosen and why – page 10
Statistical methods	12	(*a*) Describe all statistical methods, including those used to control for confounding – page 10
		(*b*) Describe any methods used to examine subgroups and interactions – page 10
		(*c*) Explain how missing data were addressed N/A
		(*d*) If applicable, explain how matching of cases and controls was addressed - page 10
		(*e*) Describe any sensitivity analyses – page 10
**Results**		N/A
Participants	13*	(a) Report numbers of individuals at each stage of study—eg numbers potentially eligible, examined for eligibility, confirmed eligible, included in the study, completing follow-up, and analysed N/A
		(b) Give reasons for non-participation at each stage N/A
		(c) Consider use of a flow diagram – N/A
Descriptive data	14*	(a) Give characteristics of study participants (eg demographic, clinical, social) and information on exposures and potential confounders N/A
		(b) Indicate number of participants with missing data for each variable of interest N/A
Outcome data	15*	Report numbers in each exposure category, or summary measures of exposure N/A
Main results	16	(*a*) Give unadjusted estimates and, if applicable, confounder-adjusted estimates and their precision (eg, 95% confidence interval). Make clear which confounders were adjusted for and why they were included N/A
		(*b*) Report category boundaries when continuous variables were categorized N/A

For peer review only - http://bmjopen.bmj.com/site/about/guidelines.xhtml

		(*c*) If relevant, consider translating estimates of relative risk into absolute risk for a meaningful time period N/A
Other analyses	17	Report other analyses done—eg analyses of subgroups and interactions, and sensitivity analyses – N/A
**Discussion**		
Key results	18	Summarise key results with reference to study objectives – N/A
Limitations	19	Discuss limitations of the study, taking into account sources of potential bias or imprecision. Discuss both direction and magnitude of any potential bias - page 3
Interpretation	20	Give a cautious overall interpretation of results considering objectives, limitations, multiplicity of analyses, results from similar studies, and other relevant evidence – N/A
Generalisability	21	Discuss the generalisability (external validity) of the study results – N/A
**Other information**		
Funding	22	Give the source of funding and the role of the funders for the present study and, if applicable, for the original study on which the present article is based – page 11/12

*Give information separately for cases and controls.

Note: An Explanation and Elaboration article discusses each checklist item and gives methodological background and published examples of transparent reporting. The STROBE checklist is best used in conjunction with this article (freely available on the Web sites of PLoS Medicine at http://www.plosmedicine.org/, Annals of Internal Medicine at http://www.annals.org/, and Epidemiology at http://www.epidem.com/). Information on the STROBE Initiative is available at http://www.strobe-statement.org.

For peer review only

For peer review only

For peer review only - http://bmjopen.bmj.com/site/about/guidelines.xhtml
